# Interactions of HIV and Antiretroviral Therapy With Neutrophils and Platelets

**DOI:** 10.3389/fimmu.2021.634386

**Published:** 2021-03-12

**Authors:** Morris Madzime, Theresa M. Rossouw, Annette J. Theron, Ronald Anderson, Helen C. Steel

**Affiliations:** Department of Immunology, Faculty of Health Sciences, University of Pretoria, Pretoria, South Africa

**Keywords:** neutrophils, platelets, HIV-infection, antiretroviral therapy, neutrophil-platelet aggregates

## Abstract

Neutrophils are important components of the innate immune system that mediate pathogen defense by multiple processes including phagocytosis, release of proteolytic enzymes, production of reactive oxygen species, and neutrophil extracellular trap formation. Abnormalities of neutrophil count and function have been described in the setting of HIV infection, with the majority of antiretroviral agents (ARVs), excluding zidovudine, having been reported to correct neutropenia. Questions still remain, however, about their impact on neutrophil function, particularly the possibility of persistent neutrophil activation, which could predispose people living with HIV to chronic inflammatory disorders, even in the presence of virally-suppressive treatment. In this context, the effects of protease inhibitors and integrase strand transfer inhibitors, in particular, on neutrophil function remain poorly understood and deserve further study. Besides mediating hemostatic functions, platelets are increasingly recognized as critical role players in the immune response against infection. In the setting of HIV, these cells have been found to harbor the virus, even in the presence of antiretroviral therapy (ART) potentially promoting viral dissemination. While HIV-infected individuals often present with thrombocytopenia, they have also been reported to have increased platelet activation, as measured by an upregulation of expression of CD62P (P-selectin), CD40 ligand, glycoprotein IV, and RANTES. Despite ART-mediated viral suppression, HIV-infected individuals reportedly have sustained platelet activation and dysfunction. This, in turn, contributes to persistent immune activation and an inflammatory vascular environment, seemingly involving neutrophil-platelet-endothelium interactions that increase the risk for development of comorbidities such as cardiovascular disease (CVD) that has become the leading cause of morbidity and mortality in HIV-infected individuals on treatment, clearly underscoring the importance of unraveling the possible etiologic roles of ARVs. In this context, abacavir and ritonavir-boosted lopinavir and darunavir have all been linked to an increased risk of CVD. This narrative review is therefore focused primarily on the role of neutrophils and platelets in HIV transmission and disease, as well as on the effect of HIV and the most common ARVs on the numbers and functions of these cells, including neutrophil-platelet-endothelial interactions.

## Introduction

Infection of individuals with the human immunodeficiency virus (HIV) is characterized by persistent coinfection with opportunistic microbes. Some of the most common causative pathogens include: *Mycobacterium tuberculosis, Mycobacterium avium, Streptococcus pneumoniae*, hepatitis B virus (HBV), hepatitis C virus (HCV), cytomegalovirus (CMV), *Pneumocystis jirovecii*, and *Cryptococcus neoformans* (1). Although treatment with combined antiretroviral therapy (cART) has reduced the incidence of opportunistic infections in these individuals, they remain a major cause of morbidity and mortality (2). While the related immunodeficiency is largely due to the loss of cell-mediated immunity associated with the targeted cluster of differentiation (CD) 4+ T-lymphocytes and monocytes, other immune cells, including those of the innate immune system, have also been shown to be functionally impaired in HIV-infected individuals (3).

Neutrophils are considered the first line of defense against invading microorganisms, particularly bacterial and fungal pathogens, while the importance of neutrophils in containing and eliminating viral infections is also being increasingly accepted (4, 5). Despite being recognized as mediators of hemostasis and thrombosis, the relevance of platelets in driving immune responses is now well-established. Platelets have been shown to possess antimicrobial activity against bacteria, viruses, fungi and protozoa (6), with the role platelets play in innate and adaptive immune responses having been well-documented by a number of authors (7–11).

In addition to the activation, regulation, and function of cells of the innate and adaptive immune systems being necessary for an effective immune response, the distribution and retention of these cells at sites of infection are equally important. In this context, endothelial cells interact with immune cells to facilitate these functions via formation of leukocyte:platelet heterotypic aggregates or via endothelial-leukocyte-platelet interactions, as reviewed by Danese et al. (12).

There are, however, important, albeit unanswered, questions about the kinetics and functionality of neutrophils and platelets during the course of HIV infection and how these factors impact on both HIV-specific and broader antimicrobial responses in untreated and treated individuals (13). This is further complicated by the fact that different antiretroviral (ARV) drugs impact differently on neutrophil and platelet functions by mechanisms that vary, even within the same class (13). To complicate matters even further, the use of ARVs may lead to enhanced, reduced, or dysregulated interaction between neutrophils and platelets, which, in turn, may attenuate or exacerbate the progression of the disease. Evaluating the effects of different ARVs, alone and in combination, on neutrophil and platelet activation, as well as on the interaction of the two with the endothelium, would enable valuable insights into the roles these cells play in the immunopathology of HIV, potentially opening up new avenues for treatment.

This review discusses the role played by neutrophils and platelets in HIV transmission and disease and the effect of HIV and the most common ARV agents on the numbers and functions of these cells, as well as on neutrophil-platelet interactions. It concludes with a brief discussion of the effect of HIV and ART on neutrophil-platelet-endothelium interactions and the implications of these for development of CVD.

## Neutrophils

### The Role of Neutrophils in HIV Transmission and Disease

Neutrophils comprise 50–70% of all circulating leukocytes and play an important role in protecting the host from invading infectious pathogens. They contain cytoplasmic granules, which are comprised of various antimicrobial peptides and proteins that facilitate the breakdown and killing of internalized microbes. These antimicrobial mediators include defensins, cathepsins, proteinase-3, elastase, azurocidin, and lysozymes (14). Flavocytochrome *b*_558_ and lactoferrin are also released during degranulation of the cytoplasmic granules (14). The role of these antimicrobial agents has recently been reviewed by Kobayashi et al. (14). In addition, neutrophils produce superoxide via the nicotinamide adenine dinucleotide phosphate (NADPH) oxidase complex. Superoxide, in turn, is converted to other reactive oxygen species (ROS), such as hydrogen peroxide and hypochlorous acid, catalyzed by superoxide dismutase and myeloperoxidase, respectively (15). These ROS are efficient oxidizing agents and have an even greater ability to kill phagocytosed microbes (16). If excessive, the release of neutrophil-derived ROS, proteases and other effector molecules may, however, result in collateral damage to surrounding tissue in the vicinity of acute and chronic inflammatory responses (17).

The transmission and pathogenesis of HIV has been reported to be influenced by neutrophils. Neutrophils have been found to bind HIV-1 and to transfer the virus to T-lymphocytes, thereby increasing the risk of HIV transmission, particularly at the mucosal interface (18). In this context, it was observed that binding of the virus to neutrophils occurred independently of CD4 or gp120 (19) and that the HIV bound to these cells was ~9-fold more infectious than the same amount of free HIV-1 (18). It was suggested that the enhanced infectivity of neutrophil-bound virus was not due to stimulatory signals provided, or by either infection of the cells or via selection of infectious particles, as observed in another CD4-negative cell type, the Raji cells (18). Nevertheless, further investigations are necessary to confirm these findings and to determine potential mechanisms of augmentation of infectivity (18).

In contrast, the recruitment and activation of other immune cells involved in the control and elimination of HIV are also initiated by the release of proinflammatory mediators from HIV-adherent neutrophils, with α-defensins 1–4 having been shown to inhibit the replication of HIV-1 *in vitro* (20).

HIV infection is characterized by the continued loss of CD4+ T-lymphocytes and an imbalance in CD4+ T-lymphocyte homeostasis, which leads to a gradual loss of immune functionality that occurs in the setting of chronic immune activation (21). Neutrophils have been implicated in the pathogenesis of T cell dysfunction in HIV infection. This contention is supported by a study reported by Bowers et al., who found that neutrophils in the blood of HIV-infected individuals expressed high levels of programmed death-ligand 1 (PD-L1) that correlated with the expression of programmed death-1 (PD-1) and CD57 on CD4+ and CD8+ T-lymphocytes (22). These authors noted that one of the mechanisms by which neutrophils isolated from HIV-infected individuals inhibited the function of T-lymphocytes involved PD-L1/PD-1 interaction and production of ROS by neutrophils which, in turn, contributed to the ongoing T-lymphocyte exhaustion and immune suppression observed in HIV-infection, clearly favoring viral persistence (22). Importantly, PD-L1 expression by neutrophils, as well as monocytes, was significantly reduced following initiation of cART (22). These findings appear to support the therapeutic use of PD-1/PD-L1-targeted monoclonal antibodies as a strategy to attenuate neutrophil-mediated T cell dysfunction in the setting of HIV infection.

### The Effect of HIV on Neutrophils

HIV-infected individuals have been reported to present with decreased counts of circulating neutrophils (neutropenia) compared to their uninfected counterparts with neutrophil counts dropping further with disease progression (23–25). HIV-mediated cytotoxicity of progenitor cells and other leukocytes is considered to contribute to the observed neutropenia through reduced neutrophil production in the bone marrow (13). Of note, however, is that to our knowledge, no studies to date have demonstrated that HIV directly infects and kills mature neutrophils (13, 24). Loss of peripheral neutrophils in HIV-infected individuals may also be due to increased spontaneous apoptosis of these cells (26, 27). HIV proteins modulate ROS *in vitro*: the HIV trans-activator of transcription protein (Tat) increases H_2_O_2_ production which mediates activation-induced cell death, while env gp41 increases ROS in bystander cells, facilitating env-mediated apoptosis (28). Other mechanisms involved in the apoptosis of neutrophils include increased sensitivity to Fas-induced apoptosis and accumulation and/or increased calpain activity in HIV-infected individuals (29, 30). Similar findings were reported in simian immunodeficiency virus (SIV)-infected Asian Rhesus macaques (31). For a comprehensive review of other mechanisms of neutropenia associated with HIV-infection, including autoimmune disorders, bone marrow toxicity of secondary infections and the hemophagocytic syndrome, readers are referred to the article by Shi et al. (24).

Abnormalities of neutrophil function, such as impaired neutrophil development, as well as compromised adhesion, chemotaxis and recruitment, have been reported in HIV-infected individuals (28, 32, 33). As discussed below, these compromised neutrophil functions have generally been shown to improve with cART-treatment, specifically with regimens containing protease inhibitors (PIs) (34). However, impaired phagocytosis, which persists despite cART treatment, has also been reported (35). Several mechanisms for deficient phagocytosis in HIV-infected individuals have been suggested, including compromised opsonization and defective formation of the phagosome (36, 37). In addition, neutrophils from HIV-infected individuals also exhibit effector defects such as reduced cytokine production, impaired degranulation, reduced responses to endotoxin stimulation, downregulation of the metabolism of ROS and impaired antibody-mediated cytotoxicity (31). Depressed neutrophil function in these individuals most likely contributes to frequent co-infections, as well as to increased disease progression. These effects on neutrophils have been shown to improve following initiation of cART (38).

In contrast to systemic apoptosis of neutrophils, Hensley-McBain et al. have recently reported that there is increased survival of neutrophils in the gastrointestinal tract (GIT) of HIV-infected individuals, seemingly due to reduced neutrophil apoptosis as a consequence of microbial dysbiosis. Reduced apoptosis may, in turn, lead to the accumulation of GIT neutrophils, thereby contributing to mucosal inflammation/damage and increased microbial translocation that persists, despite long-term cART (39). In addition, attenuation of apoptosis may lead to a delay in the adaptive immune response, as has been reported for other pathogens, due to fewer apoptotic vesicles ingested by antigen presenting monocytes and dendritic cells (DCs) (40), as well as increased release of pro-inflammatory cytokines and chemokines (41).

An aging neutrophil phenotype has been described as these cells progress through various stages of development with each stage having defined functions and properties (42). Increased survival of neutrophils, up to 5.4 days, has been reported for inflammatory conditions, as well as for other chronic viral infections, such as CMV (43, 44). A study determining the maturation state of neutrophils in a SIV-macaque non-human primate infection model, revealed that the later stages of chronic SIV infection were associated with an immature neutrophil phenotype with mature neutrophils no longer present in the blood (45), seemingly indicating apoptosis of aged neutrophils, or mobilization of these cells to the tissues where viral replication sustains inflammation (45). These findings were consistent with the fact that CXCR4 expression decreases in young circulating neutrophils, while aged neutrophils re-express CXCR4 allowing them to home to specific sites and to be cleared by macrophages in the spleen and bone marrow (45–47). Although, in limited studies, aged neutrophils have not been demonstrated to be of significant etiologic importance in SIV, their role, if any, remains to be established in the human setting of HIV infection.

**Figure 1:**
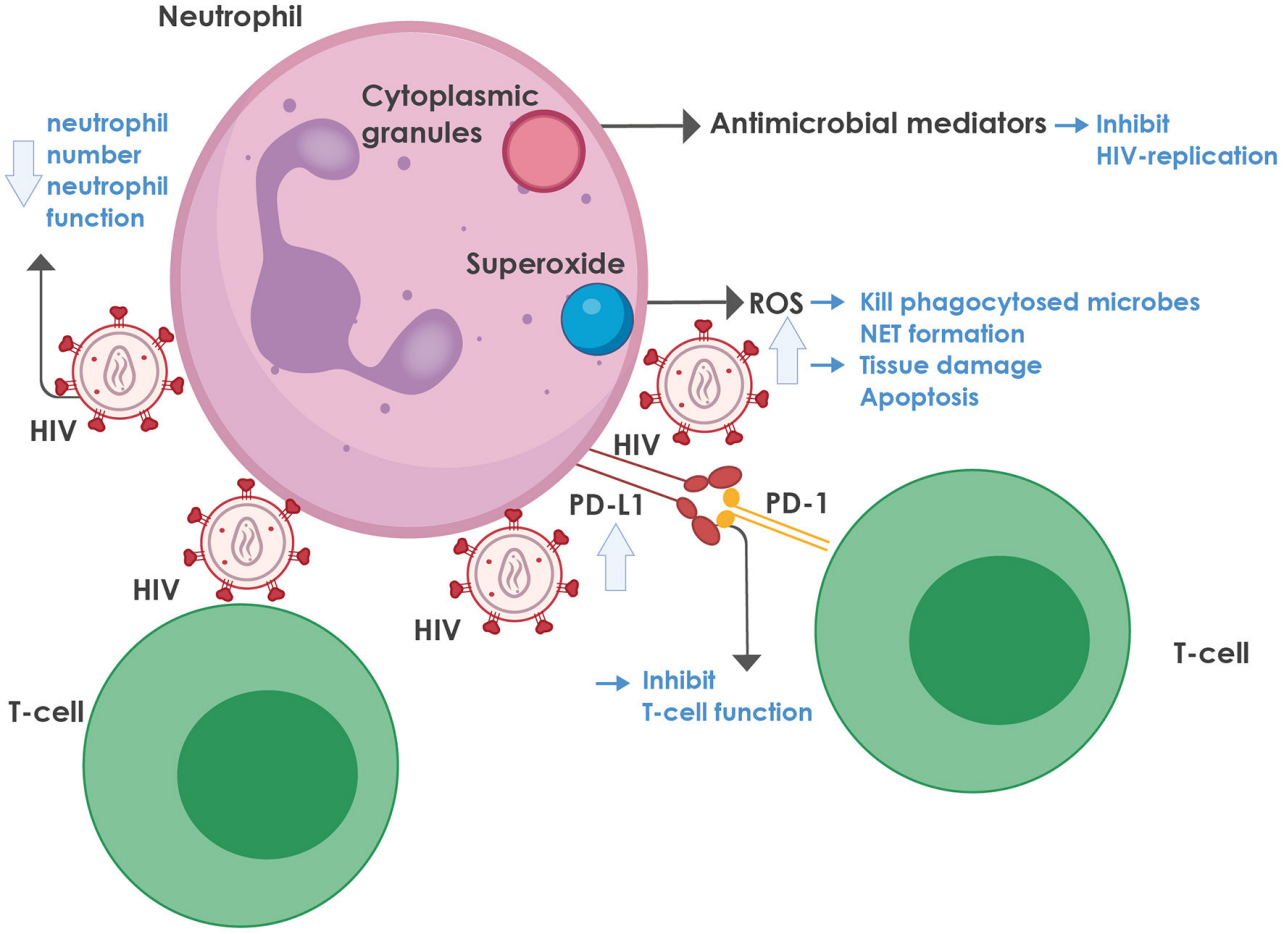
The role of neutrophils in HIV infection. Neutrophils contain cytoplasmic granules, consisting of various antimicrobial mediators, notably defensins, that inhibit HIV replication. In addition, neutrophils produce superoxide, which is converted to reactive oxygen species (ROS) that can kill phagocytosed microbes and mediate neutrophil extracellular trap (NET) formation. On the downside, ROS also lead to tissue damage and activation-induced cell death. ROS production is enhanced during HIV infection, mediated by various viral proteins, most notably the envelope glycoprotein, gp120 and the regulatory protein, trans-activator of transcription protein (Tat), as well as by the regulatory proteins, negative regulating factor (Nef), virus protein r (Vpr), and reverse transcriptase. HIV inhibits both the number and function of neutrophils in the blood through multiple mechanisms (more detail is provided in the text). HIV-infected individuals also express high levels of programmed death-ligand 1 (PD-L1) that binds to programmed death-1 (PD-1) on T-cells, thereby contributing to T-cell dysfunction and exhaustion. NET, neutrophil extracellular trap; PD-1, programmed death-1; PD-L1, programmed death-ligand 1; ROS, reactive oxygen species.

For a summary of the role of neutrophils in HIV infection, see Figure 1.

The currently available evidence is consistent with a suppressive effect of HIV infection on the numbers of circulating neutrophils that seemingly results from a combination of several mechanisms most prominently neutropenia associated with progressive disease, as well as infection-related pro-apoptotic mechanisms, predisposing for development of secondary bacterial infection.

### The Effect of Antiretroviral Agents on Neutrophils

The most commonly used ARV agents belong to four classes: (i) nucleoside and nucleotide reverse transcriptase inhibitors (NRTIs and NtRTIs); (ii) non-nucleoside reverse transcriptase inhibitors (NNRTIs); (iii) protease inhibitors (PIs); and integrase strand transfer inhibitors (INSTIs) (48). Each is discussed below with regard to their effects on neutrophils and platelets, as well as interactions between these two cell types, followed by a discussion of the effect of these agents when combined as cART.

While other new drug classes, such as entry and fusion inhibitors are also available, only two members of these classes of ARVs have been approved for clinical use. Since these classes are mostly still restricted to clinical trials and specialized treatment centers with limited “real-world” experience, they are not discussed in this review.

#### Nucleos(t)ide and Non-nucleoside Reverse Transcriptase Inhibitors

NRTIs interrupt HIV replication by inhibiting the reverse transcriptase enzyme. Shortly after the first NRTI, zidovudine (ZDV), also known as azidothymidine (AZT), was approved for the treatment of HIV, several studies revealed that the drug had effects on neutrophil production (49). Over the years, zidovudine, either alone, or in combination with other ARV agents, has been demonstrated to cause neutropenia (50–53). Seemingly in agreement with these findings, a study conducted in HIV-infected women receiving cART, which did not include zidovudine in the regimen, was associated with protection against neutropenia (51). In this context, investigation of the hematological profiles of a cohort of individuals receiving cART at a Nigerian teaching hospital revealed higher frequencies of leukopenia and neutropenia (52). Another study in West Africa also reported that HIV-infected persons initiating a cART regimen that included zidovudine, exhibited severe neutropenia associated with advanced HIV disease (25). These adverse effects of zidovudine result from bone marrow toxicity and myelosuppression (54).

In addition to suppressing neutrophil production, ARV drugs may also affect neutrophil function. In this context, an early study by Roilides et al. examined the *in vitro* effects of ARV dideoxynucleoside agents [dideoxyinosine [didanosine], dideoxycytidine [zalcitabine], and zidovudine] on the function of neutrophils from healthy control and HIV-infected participants. Didanosine and zalcitabine, but not zidovudine, enhanced the killing of *Candida albicans* and *Staphylococcus aureus*, by neutrophils from both groups, while none of the test agents affected neutrophil viability, chemotaxis, phagocytosis, or superoxide production in response to N-formyl-methionyl-leucyl-phenylalanine (FMLP) (55). Another earlier study by Pitrak et al. reported that zidovudine therapy was associated with impaired neutrophil oxidative metabolism in HIV-infected patients, but conceded that these effects of the drug may have reflected a longer duration of disease relative to the comparator group of treatment-naïve patients (56). Indeed, these investigators were unable to detect suppressive effects of this drug on superoxide production by neutrophils *in vitro* (56).

#### Protease Inhibitors

PIs are peptide-like substrate analogs that bind to the protease active site and interrupt the HIV lifecycle by blocking the viral enzyme, HIV aspartyl protease, which is responsible for the proteolytic cleavage of the HIV Gag and Gag-Pol polyprotein precursors into mature active proteins (57). PIs have been reported to improve the clinical status and immune function of HIV-infected individuals in the absence of an antiviral effect (58) by means of extra-virologic properties that affect cellular turnover and metabolism. Many processes may be involved, but the best studied mechanism explaining how PIs restore normal white cell counts in HIV-infected persons is through inhibition of apoptosis in CD4+ T-lymphocytes.

There are two major apoptotic pathways, namely the extrinsic, death-receptor pathway, which is triggered through ligation of death receptors that activate the caspase family, and the intrinsic BCL-2 regulated mitochondrial pathway, which is set in motion by mitochondrial injury (59). The exact mechanisms leading to the anti-apoptotic effects of PIs are still under investigation, but both the extrinsic and intrinsic pathways have been implicated. While PIs do not inhibit the serine protease family of caspases, they have been shown to inhibit the calcium-dependent cysteine protease, calpain, which plays an important role in apoptosis (60). The second mechanism is through the maintenance of mitochondrial integrity, possibly by inhibiting pore formation by the adenine nucleotide translocator subunit of the mitochondrial permeability transition pore complex (61). Another proposed mechanism involves decreasing basal susceptibility to apoptosis by inhibiting the entry of lymphocytes into the cell cycle (62). The effect of PIs on other leukocytes is less well-studied, but an anti-apoptotic effect is believed to play a role in the restoration of neutrophil counts (63). *In vitro* and *ex vivo* experiments have demonstrated an inhibitory effect on μ-calpain, a neutrophil cysteine protease that has been reported to play a role in spontaneous apoptosis as mentioned above (30, 64).

While treatment with PIs leads to an improvement in neutrophil counts, their impact on neutrophil function is variable. A small study of eight individuals demonstrated increased shedding of L-selectin (CD62L) when compared to the HIV-negative reference range in response to *in vitro* FMLP stimulation after initiation of PI-based therapy. Since CD62L shedding is important for neutrophil extravasation to proceed, this finding suggests that PI-based therapy improves the chemotactic function of neutrophils (65). In contrast, Hadad et al. demonstrated that PIs directly inhibit various neutrophil functions, namely superoxide production [after stimulation with phorbol myristate acetate [PMA], FMLP, and opsonized zymosan], chemotaxis, and phagocytosis *in vitro*. Interestingly, these inhibitory effects coincided with the anti-calpain activity of PIs and occurred at concentrations readily achievable with oral treatment regimens. PIs differed in their ability to impair neutrophil function, with saquinavir and nelfinavir being the most effective, followed by lopinavir and ritonavir, while amprenavir was the least potent (64). This differential effect coincided with the intracellular drug concentrations achieved by each drug, as determined by differences in their uptake mechanisms, affinities and accumulation within peripheral blood mononuclear cells (66, 67).

#### Integrase Strand Transfer Inhibitors

INSTIs bind to the catalytic core domain of the viral integrase to inhibit binding of the enzyme to the double-stranded DNA of the host. Since no equivalent homologs of integrase are known to exist in humans, INSTIs are generally believed to have limited off-target effects (68). Recent computational work, however, demonstrated that INSTIs could potentially interact with domesticated transposases, such as recombination activating gene 1 (RAG1), which is known to be involved in antibody and T-lymphocyte receptor V(D)J recombination. Retroviral integrase and RAG1 both belong to the DDE polynucleotidyl transferases superfamily and have similar mechanistic and structural characteristics related to catalytic domain organization and DNA cleavage. It is therefore possible that INSTIs cause non-specific inhibition of RAG1, resulting in aberrant immune receptors (69). Previous studies have linked the use of INSTIs to an increased risk of non-Hodgkin's lymphoma (NHL) (70, 71). Notwithstanding the possibility that NHL could be secondary to Epstein Barr virus co-infection, other HIV-1 induced oncogenic mechanisms, severe immunosuppression, immune reconstitution inflammatory syndrome, or enhanced cytokine responses (72), the possibility exists that alterations in RAG1 recombination could be responsible for the proliferation of abnormal lymphocytes (70).

It is fascinating to speculate how RAG1 inhibition might affect neutrophils. Traditionally classified as part of the innate immune system, studies have, however, revealed the presence of a T-cell receptor-based variable immunoreceptor in a subpopulation of neutrophils (73). Interestingly, neutrophils constitutively express RAG1, implying that they have the ability to generate antigen receptor diversity. Activation of this receptor by T-lymphocyte receptor agonists inhibits neutrophil apoptosis (73), however, it is unknown whether inhibition of RAG1 facilitates apoptosis. While neutropenia has been reported in some cases of RAG1-deficiency, they seem to be the consequences of auto-immune processes driven by abnormal T- and B-lymphocyte tolerance (74) rather than an intrinsic neutrophil defect. While neutropenia is not a common side-effect of INSTIs, decreases in absolute neutrophil counts have been reported in clinical trials and the mechanisms involved deserve further study (75).

#### Combination Antiretroviral Therapy

Apart from zidovudine-containing regimens being associated with neutropenia due to their myelosuppressive actions, other cART regimens have generally been reported to resolve neutropenia in HIV-infected individuals with improvement in CD4+ T-lymphocyte counts following successful viral suppression (76). A study by Campillo-Gimenez et al. found increased basal hyperactivation of neutrophils in HIV-infected individuals, even in the absence of inflammatory disease. However, neutrophil hyperactivity was higher in those individuals with inflammatory diseases (77). The observed hyperactivation may, in turn, be associated with a switch in the equilibrium between apoptosis and necrosis in these individuals and was found to persist even in those individuals receiving cART (77). This imbalance was accounted for by impaired phagocytosis of apoptotic neutrophils by macrophages isolated from HIV–infected patients (78) and may, in turn, play a role in the increased risk of HIV-infected individuals to develop chronic inflammatory disorders such as cardiovascular disease (CVD) and osteoarticular conditions (77).

Combination ART has also generally been reported to improve neutrophil function. A combination of the PIs, indinavir or ritonavir, and two NRTIs improved neutrophil and monocyte function in HIV-infected patients (34). The authors reported that these individuals showed diminished baseline chemotactic and fungicidal activity compared to healthy controls (34). Following initiation of cART, there was an improvement of these activities of phagocytic cells. In addition, administration of cART was also associated with an enhanced oxidative burst by both neutrophils and monocytes, as measured by increased chemiluminescence responses following stimulation with PMA or opsonized *Candida albicans* (34). These findings were confirmed by those of a more recent study showing that cART, concomitant with an increase in CD4 counts and a decrease in HIV viral load, improved neutrophil and monocyte phagocytosis and the oxidative burst in HIV-infected persons (33).

Another earlier study reported that isolated blood neutrophils from HIV-infected patients with high viral loads had a selective defect of antimicrobial activity against the encapsulated strain of *Cryptococcus neoformans* (79). This abnormality of neutrophil antimicrobial activity, as well as the findings of a dysregulated respiratory burst and anomalous release of IL-12 by unstimulated neutrophils, all normalized after 3 months of cART that included at least three NRTIs (didanosine, zidovudine, stavudine, lamivudine) and one PI (indinavir, ritonavir) (79). In apparent contrast, however, Tsachouridou et al. reported a downward trend in neutrophil phagocytic activity over a 48-weeks period in HIV-1 infected adults, irrespective of intake of an unspecified cART regimen. The authors suggested that the defect stemmed from persistent abnormalities in the Fc gamma phagocyte receptor, both in recognition of the microorganism and intracellular signaling (35). The differences observed in these studies could be explained by different participant characteristics, disease stage, concurrent infections and medication, as well as methodological differences and the use of non-standardized assays.

Notwithstanding the detrimental effect of suboptimal phagocytic function, excessive or inappropriate production of antimicrobial substances, such as ROS, also have harmful effects (80). For example, in a study undertaken to evaluate the chronic effects of cART (tenofovir + emtricitabine + atazanavir/ritonavir) on oxidative stress and cardiac dysfunction in HIV-1-infected transgenic rats, it was found that cART increased basal, but not PMA-stimulated superoxide production by isolated neutrophils (81). These effects, which were also previously described in the context of monotherapy with zidovudine or ritonavir, could potentially contribute to cardiac toxicity and were attenuated by manganese supplementation as an anti-oxidative strategy to augment superoxide dismutase (82, 83).

While the effects of INSTIs and new drug classes on the numbers of circulating neutrophils and their functions remain to be established, the effects of administration of other ARVs, with the exception of zidovudine, on restoration of neutrophil counts in advanced HIV infection are clearly beneficial. For most of these agents, attenuation of HIV-associated neutropenia is probably achieved via recovery of bone marrow hematopoietic activity, while the anti-apoptotic activity of PIs may also contribute. The effects of ARVs on neutrophil functions are, on the other hand, somewhat variable and of uncertain clinical relevance.

The effect of HIV on neutrophil function, the mechanisms involved, and the effects of cART on these functions are summarized in Table 1.

**Table 1:** The effects of HIV-infection and combination antiretroviral therapy on the effector functions and numbers of neutrophils.

**Effector function of neutrophils (4, 84)**	**Effect of HIV on neutrophil function and number**	**Consequence**	**Effect of cART on neutrophil function**
	• Neutropenia (51, 85, 86). • Binding of virus to neutrophils resulting in infection of T-lymphocytes (19).	• Increased HIV transmission. • Disease progression (SIV) (87).	• Improved neutrophil counts with increase in CD4+ T-lymphocyte counts (51).
• NET formation • Release of proteolytic enzymes and peptides (elastase, α-defensins, cathepsin G, cathelicidins).	• Induces NET formation (87–89). • Reduction in antimicrobial enzymes and peptides [α-Defensins (90–92), Myeloperoxidase (93)].	• Decreased protection against HIV transmission.	• Decreases NETosis albeit above pre-infection levels (87, 88)
• NADPH oxidase and myeloperoxidase activity. • ROS production.	• Release of ROS by neutrophils (4, 54). • Release of granular proteins and peptides produced by neutrophils (4).	• Mucosal dysfunction of the GIT, secondary to tissue damage. • Increased microbial translocation due to mucosal damage (94–96).	• Mucosal barrier functions remain impaired (97).
• Immunomodulatory activity (87).	• Increased expression of PD-L1 by neutrophils (22).	• Suppressed T-lymphocyte function and T-lymphocyte exhaustion causing immune suppression (22, 98).	• Reduced PD-L1 expression by neutrophils (22).
• Chemotaxis and immune cell recruitment. • Phagocytosis. • Cytokine and chemokine production.	• Neutropenia (99) due to increased peripheral neutrophil apoptosis, calpains (64). • Impaired CD62L, increased CD11b and CD18 expression (100). • Reduced chemotaxis and recruitment (32, 101). • Reduced phagocytosis (26, 33). • Reduced production of granular proteins and peptides (90). • Reduced ROS production (33). • Reduced cytokine production (102, 103).	• Increase in secondary infections.	• Improved neutropenia with increase in CD4+ T-lymphocyte counts (51). • Improved chemotaxis (34), phagocytosis (34, 102), ROS production (33, 34). • Reduction in apoptosis (51).

*cART, combined antiretroviral therapy; NET, neutrophil extracellular trap; NETosis, neutrophil extracellular trap activation and release; SIV, simian immunodeficiency virus; NADPH, nicotinamide adenine dinucleotide phosphate; ROS, reactive oxygen species; PD-L1, programmed death-ligand 1; CD, cluster of differentiation*.

## Platelets

### The Role of Platelets in HIV Transmission and Disease

As with neutrophils, platelets are able to trap and eliminate pathogenic microorganisms (14, 104–107). The interaction of pathogens with platelets is well-recognized with bacteria, parasites and viruses binding to platelets via receptors expressed on their surface, resulting in platelet activation (8, 106, 108). In addition to pathogen recognition receptors, platelets also possess an array of different receptors and ligands that promote interaction with various cells of the innate and adaptive immune systems, as well as with structural cells (11, 109). With respect to antimicrobial activity, platelets possess an array of mediators, including granule-derived antimicrobial peptides such as defensins, as well as cytokines, chemokines, and adhesion molecules. Chemokine receptors present on platelets result in activation of neighboring platelets and other cells of the innate and adaptive immune systems with the release of CD40 ligand (CD40L) from activated platelets coordinating adaptive immune responses (110).

Platelets have also been shown to interact with HIV, leading to the internalization of the virus. HIV-infection of platelets has been proposed to occur as a result of active thrombopoiesis of megakaryocytes that have been infected with the virus (111). An alternative theory of spontaneous HIV uptake by platelets has been suggested due to platelets expressing dendritic cell-specific intercellular adhesion molecule-3 (ICAM3)-grabbing non-integrin (DC-SIGN) and C-type lectin receptor 2 (CLEC-2) (111, 112). Uptake of HIV by platelets may play a role in host defense against the virus by preventing dissemination of the virus, as well as inactivating viral particles (106, 107). It is noteworthy that Regulated on Activation, Normal T Cell Expressed and Secreted (RANTES), produced and secreted by platelets, may inhibit the entry of HIV into cells by binding to the HIV co-receptor CCR5 (113). However, platelets, as well as their precursors, megakaryocytes, have been found to harbor the virus, possibly protecting it from the host immune system and acting as a reservoir for subsequent HIV infection of macrophages (114–116). Notably, HIV remains detectable in platelets for at least 3 months after initiation of cART (116). In contrast to the above reports, it has been suggested that platelets may also play an important role in promoting the dissemination of the virus in HIV-infected individuals (116). The spread of HIV by platelets to CD4+ T-lymphocytes and monocytes may occur via the P-selectin glycoprotein (GP) ligand (PSGL)-1 and P-selectin (CD62P) complex. The interaction of PSGL-1 on CD4+ T-lymphocytes and monocytes with CD62P on activated platelets occurs in a calcium-dependent manner and results in the formation of leukocyte–platelet aggregates, which are found with increased frequency in HIV-infected individuals (116, 117).

Notably, platelet activating factor (PAF), which is produced by various cell types, including platelets, is a potent mediator of inflammation. Low levels of PAF are constitutively produced by immune cells, however, when activated, inflammatory cells produce PAF in larger quantities (118, 119). PAF mediates intercellular interactions by binding to extracellular receptors of other cells, resulting in their activation and can act as a double-edged sword by reducing infection or promoting chronic inflammation (120). In platelets, PAF is responsible for platelet aggregation and facilitates the release of mediators of platelet inflammatory reactivity. Increased levels of PAF biosynthesis have been associated with disease progression in HIV-infected individuals and a mechanistic link between the PAF pathway and HIV-infection, systemic inflammation and immune activation has been suggested (121). This, in turn, may contribute to an increased risk of CVD and HIV-associated neurocognitive disorders (HAND) (122).

### The Effect of HIV on Platelets

Thrombocytopenia is readily observed in HIV-infected individuals, either as a result of increased peripheral platelet clearance of activated platelets or reduced platelet production from infected megakaryocytes (123). This also impacts on the ability of the immune system to eliminate invading pathogens as platelets express Toll-like receptors (TLRs), which, when activated by pathogen-associated molecular patterns (PAMPs) as well as damage-associated molecular patterns (DAMPs), result in the release of immune mediators that act on both the pathogen and surrounding cells to facilitate clearance. In addition, platelets are able to “collect” and encapsulate invading microorganisms, thereby preventing dissemination of the microbes (6). This, in turn, leads to increased activation of recruited phagocytes, as well as the release of neutrophil extracellular traps (NETs) (124).

Increased platelet activation is observed in HIV-infected individuals and an association between HIV-1 and platelets has been established through all stages of infection with the virus binding to the platelets via a number of receptors, including TLRs. Internalized virus may also interact with endosomal TLR7 as well as with TLR9 in T-granules, leading to increased platelet activation (125). Most notably, platelet granules express CXCR1, 2, 4, and CCR3 co-receptors that are used for interaction with HIV-1 (106). Moreover, HIV-1 Tat directly interacts with platelets via CCR3 and β3-integrin, resulting in platelet activation and the release of platelet microparticles and the pro-inflammatory adhesion receptor, CD40L (6). In addition, as mentioned above, platelets also express DC-SIGN and CLEC-2, which facilitate the interaction of platelets with HIV-1 (112). Despite cART treatment, some studies have found that HIV-infected individuals continue to show increased levels of activated and reactive platelets (126–128), while other authors have reported a reduction in platelet hyperactivity following treatment with some regimens (128). This is discussed in more detail below.

**Figure 2:**
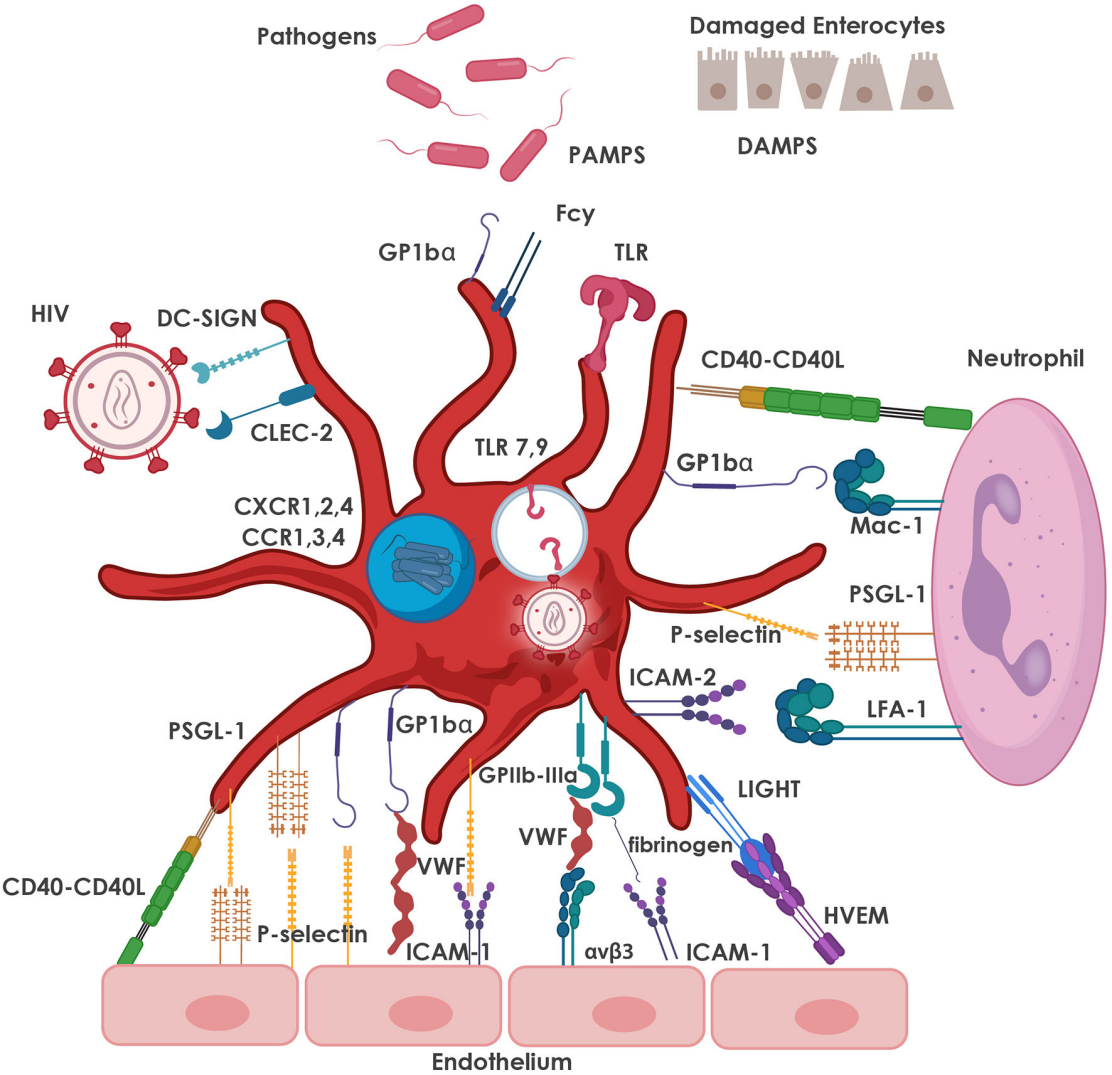
Major adhesive interactions between platelets and pathogens, including HIV, as well as with neutrophils and the endothelium. Platelets express TLRs which are activated by PAMPs and DAMPs, resulting in the release of immune mediators that facilitate pathogen clearance. Platelets also express DC-SIGN and CLEC-2 which facilitate the interaction of platelets with HIV-1. Internalized virus interacts with endosomal TLR7, as well as TLR9, leading to increased platelet activation. Platelet granules also express CXCR1, 2, 4 and CCR1, 3, 4 co-receptors that are also used for interaction with HIV-1. Neutrophil-platelet interaction is primarily mediated by the binding of CD62P on platelets to its counter-receptor, PSGL-1, on the neutrophil surface, and platelet glycoprotein (GP) Ibα binding to neutrophil Mac-1 (CD11b/CD18). Other major pro-adhesive interactions between platelets and neutrophils involve CD40 and ICAM-2 on the platelet with CD40L and LFA-1 on the neutrophil. The most relevant interactions between platelets and endothelial cells are depicted, namely platelet GPIbα (in the GPIb-IX-V receptor complex) with von Willebrand factor (VWF) released from the endothelium; bi-directional binding of CD62P with PSGL-1 or GP1bα; platelet GPIIb/IIIa (integrin αIIbβ3) with endothelial α_v_β_3_ (via VWF, fibrinogen, fibronectin), ICAM-1 (via fibrinogen); and GPIb-IX-V (via VWF). Various markers of platelet activation have been found to be upregulated in HIV-infection and include CD62P, CD40L and LIGHT (TNFSF 14). CCR, C-C chemokine receptor; CD40L; CD40 ligand; CD62P, P-selectin; CLEC-2, C-type lectin receptor 2; CXCR, C-X-C chemokine receptor; DAMPs, damage-associated molecular patterns; DC-SIGN, dendritic cell-specific soluble intercellular adhesion molecule-1-3-grabbing non-integrin; GP, glycoprotein; ICAM, intercellular adhesion molecule; LFA, leukocyte function associated antigen; Mac-1, macrophage-1 antigen; PAMPs, pathogen-associated molecular patterns; PSGL-1, P-selectin glycoprotein ligand; TLR, Toll-like receptors; TNFSF, tumor necrosis factor superfamily; VWF, von Willebrand factor.

Activated platelets mediate inflammatory and immune responses through the release of chemokines and CD62P-mediated interactions with leukocytes (129). Chronic activation of platelets from HIV-infected individuals leads to exhaustion of stored factors in platelet granules and decreased granule translocation and secretion after thrombin stimulation (130). Despite cART-mediated viral suppression, HIV-infected individuals continue to present with sustained platelet activation and dysfunction that may lead to increased comorbidities such as CVD (130), which, as mentioned above, has become one of the leading causes of morbidity and mortality in HIV-infected individuals on cART (131). The most common interactions between platelets, pathogens (including HIV), various cells of the immune system and the endothelium are depicted in Figure 2.

Various markers of platelet activation, several mentioned above, have been found to be upregulated in HIV-infection and include CD62P, CD40L, GPIV, RANTES, neutrophil activating peptide-2 (NAP-2), and LIGHT [homologous to lymphotoxin, inducible expression, competing for GpD of herpes virus, that binds to the HVEM, and is expressed on activated T-lymphocytes and platelets (also known as tumor necrosis superfamily 14)] (127, 130, 132–135). A potential mechanism of platelet activation that has been suggested by Pastori et al. is that HIV-1 mediates the induction of oxidative stress by platelet NADPH oxidase 2 (NOX2) (136).

### The Effect of Antiretroviral Agents on Platelets

The risk of CVD in HIV infection is accentuated by the use of certain combinations of ARVs (136). Emerging evidence suggests a role for persistent immune activation and dysfunction, in which platelet activation may play a key role (130).

#### Nucleos(t)ide and Non-nucleoside Reverse Transcriptase Inhibitors

Although subject to controversy (137), abacavir, an NRTI widely employed in the treatment of HIV infection, has been associated with an increased risk of myocardial infarction (MI) (138–141), with altered platelet function proposed as a potential underlying mechanism. Several studies have explored the effect of this NRTI on the induction of platelet hyperreactivity. In this context, incubation of human whole blood with abacavir, a guanosine analog, was shown to increase adenosine diphosphate (ADP)-induced platelet activation as assessed by sCD62P expression (142). A molecular mechanism for this effect was provided by the finding that the active metabolite of abacavir, carbovir triphosphate, competitively inhibited guanylyl cyclase by mimicking the natural substrate, guanosine triphosphate (GTP). This prevents the intracellular formation of cGMP (cyclic guanosine monophosphate), an essential negative regulator of platelet function (142, 143).

Satchell et al., who explored platelet function in HIV-infected patients, reported that abacavir treatment consistently caused higher percentages of platelet aggregation in response to ADP, collagen, epinephrine and thrombin receptor-activating peptide (TRAP) *ex vivo* (144). A retrospective, case-control study reported that treatment with regimens that included abacavir, but not tenofovir, was also associated with increased levels of the platelet activation markers, soluble (s) CD62P, sCD40L, secretory phospholipase A2 (sPLA2), and sGPV (145). Moreover, following oral administration of abacavir, the nitric oxide (NO)-induced increase of platelet cGMP was blunted, potentially explaining the dose-dependent enhancement in platelet aggregation (143, 145). While some reports failed to detect stimulatory effects of abacavir on platelets (146, 147), most of the aforementioned studies, including those that found that abacavir caused platelet granule release *in vitro* (148), appear to support the involvement of abacavir in the augmentation of platelet function, both *in vitro* and *in vivo*, whereas tenofovir-based therapies had no effect (148). In this context, Taylor et al. conclude that “these observations could explain epidemiological and clinical observations linking abacavir with increased incidence of platelet-driven cardiovascular events such as myocardial infarction” (148).

Interestingly, treatment with the NNRTI, efavirenz, but not abacavir plus lamivudine, was found to increase systemic levels of sCD40L in both HIV-infected individuals and in an *in vitro* suspension of washed human platelets, which is the main source of circulating sCD40L (149). The lack of effects of abacavir on sCD40L shown here was in contrast with the findings mentioned previously (145) and could reflect possible differences in methods used, or be due to other confounding factors. Elevations in sCD40L could have dire clinical consequences since CD40L has been shown to be upregulated in persons with HAND (150) possibly through aberrant activation of monocytes with subsequent neurotoxicity (150). In addition, efavirenz was also found to activate glycogen synthase kinase 3 beta (GSK3β) (149), an enzyme that has been associated with HAND (150) in platelets (149).

The platelet-to-lymphocyte ratio (PLR) is a systemic inflammatory marker that can predict distinct outcomes in different types of CVD (151). In a study undertaken between 2007 and 2015, the PLR and neutrophil-to-lymphocyte ratio (NLR) were investigated in HIV-infected patients who were switched from a tenofovir/abacavir-based triple regimen to dual therapy consisting of either a lamivudine/PI or a PI/INSTI combination (152). The PLR, but not the NLR, decreased after NRTI reduction. This trend was maintained for 12 months after tenofovir interruption, but for only 6 months after the suspension of abacavir (152). The authors concluded that more studies are needed to explore whether these fluctuations may have a clinical significance, or if they are simply a casual feature (152).

#### Protease Inhibitors

Both *in vitro* and *in vivo* studies point to potentially important interactions between PIs and PAF. As discussed above, PAF is a lipid mediator of inflammation with immunomodulatory properties that are believed to be central to the pathogenesis of inflammatory disorders, including HIV disease, where it has been suggested to play a role in the development of CVD and neurocognitive disorders (121). The PIs, saquinavir, indinavir, atazanavir, fosamprenavir, and ritonavir were found to inhibit PAF pathways *in vitro* with the latter three also reported to be weak agonists. Ritonavir-boosted lopinavir, in turn, acts as an agonist (121). Lipoprotein-associated phospholipase A_2_ (Lp-PLA_2_) is the main catabolic enzyme of PAF. Chronic activation of Lp-PLA_2_ activity increases lysophosphatidylcholine formation and hence promotes atherogenesis (122). This suggests that the long-term use of ritonavir-boosted lopinavir may increase cardiovascular risk.

In a murine model, platelets were activated by the PI, ritonavir, to produce platelet-derived transforming growth factor-β1 (TGF-β1) (153, 154). Platelet-derived TGF-β1 has been associated with the development of pathologic fibrosis and increased cardiac phosphorylative Smad signaling in murine hearts and is proposed to play a central role in the development of cardiac dysfunction (153, 154). Further *in vitro* work confirmed that ritonavir increases total TGF-β1 in a dose-dependent manner (154). At low dose, ritonavir activated latent TGF-β1 (LTGF-β1), a necessary step for signaling to occur, by four to five-fold. Atazanavir, while independently inducing release of TGF-β1 from platelets, did not activate LTGF-β1 and inhibited ritonavir-induced LTGF-β1 activation significantly. In contrast, darunavir neither independently affected TGF-β1 release and LTGF-β1 activation, nor ritonavir-induced LTGF-β1 activation (154). Darunavir has been reported to increase alpha and dense granule release in HIV-uninfected volunteers after acute exposure and it has been proposed that this may play a role in PI-related cardiovascular risk (155). Although causality cannot be claimed, these findings are consistent with clinical studies that report a progressively increased risk of CVD in HIV-infected individuals with cumulative use of ritonavir-boosted darunavir, but not with similarly boosted atazanavir (156). In fact, it has been proposed that the impact of ritonavir and darunavir on platelets could be similar to that of tobacco use (157).

Further studies of ritonavir-induced platelet activation reported increased production of prostaglandin E_2_ (PGE_2_) by platelets (158). Others similarly found that ritonavir increased platelet production of PGE_2_ in a dose-dependent fashion (159). PGE_2_ is a molecule that regulates activation, maturation, migration, and cytokine secretion by cells of the innate immune system, including neutrophils (158). In addition, ritonavir was found to increase platelet aggregation, which may be associated with increased clot strength (158, 159). Agard and colleagues suggested that ritonavir could dysregulate platelets by sensitizing them to agonists that increase PGE_2_ levels (158). Small amounts of other agonists could, therefore, make platelets hyper-responsive and lead to effector processes including neutrophil migration, neutrophil-platelet aggregation and clot strengthening (155).

#### Integrase Strand Transfer Inhibitors

In comparison to other ARVs, individuals receiving the INSTI, raltegravir, were reported to have significantly lower levels of platelet activation, as measured by expression of sCD62P on ADP stimulation, in a cross-sectional study. However, levels were still significantly higher than those of healthy volunteers (128) and a small, randomized controlled follow-up study failed to confirm these findings. In the latter study, 40 HIV-infected patients virally suppressed on non-INSTI-containing ART, were randomized to continuation therapy or switching to raltegravir. The investigators reported no change in expression of sCD62P and fibrinogen binding before and after ex-vivo stimulation with ADP, CRP-XL, and TRAP-6 at 10 weeks (160). It is, however, interesting to note that more patients in the raltegravir arm were on abacavir [raltegravir: 5 [26.3%], continuations: 3 [14.3%]], although this difference was not statistically significant (*p* = 0.342) and no baseline differences could be observed in tenofovir- vs. abacavir-treated individuals. These findings are supported by a longer-term study by Martinez et al. which reported that switching from a boosted PI to raltegravir did not reduce markers of *in vivo* platelet activation, measured by sCD62P, after 48 weeks (161).

In contrast, others have reported that switching from a ritonavir-boosted PI to raltegravir decreases Lp-PLA_2_ activity (162) and leads to a more favorable overall lipid profile (163). Also, a cross-sectional study of 80 virally suppressed patients demonstrated that HIV-infected patients on raltegravir-based ART had lower HIV-induced platelet hyperreactivity, as measured by sCD62P expression and fibrinogen binding to platelets after ADP stimulation, as well as platelet-monocyte aggregation compared with NNRTI and PI-based regimens (128). Platelet aggregation was reduced in treatment-naïve patients newly started on dolutegravir. This effect was negated when patients took dolutegravir in combination with cobicistat-boosted darunavir. In HIV-uninfected volunteers, dolutegravir decreased the release of collagen-evoked alpha and dense granules. The authors speculated that dolutegravir might confer a cardioprotective phenotype through these pathways of reduced platelet activation (155).

Integrase strand transfer inhibitors are still relatively new on the market and have, thus far, not been associated with an increased risk of CVD in the clinical setting (164, 165). While this may be related to a platelet effect, it could also be secondary to its favorable metabolic profile (166, 167) and better control of residual HIV replication (168, 169), which may also influence platelet function, since platelets are known to endocytose (91) and harbor infectious virions (170). Notwithstanding these potential cardioprotective effects, the long-term impact of INSTIs on immune cells is still poorly understood. A case of severe thrombocytopenia during dolutegravir-containing ART has also been reported by Nakaharai et al. (171). Although the mechanism was not investigated, thrombocytopenia has been reported as a common adverse event in patients receiving dolutegravir together with rilpivirine (172) and suitable surveillance is needed.

#### Combination Antiretroviral Therapy

The tenofovir/emtricitabine/efavirenz regimen was found to decrease PAF levels and metabolism *in vitro*, while tenofovir/emtricitabine together with ritonavir-boosted atazanavir as well as abacavir/lamivudine combined with either efavirenz or ritonavir-boosted atazanavir were found to increase PAF levels and metabolism *in vivo* (121, 173–175). More specifically, one research group found that, for at least the first 6 months, the abacavir/lamivudine/efavirenz regimen induces PAF biosynthesis in both neutrophils and platelets via the increased levels of lyso-PAF acetyltransferase (Lyso-PAF-AT) (173), an enzyme that is critical in the stimulus-dependent formation of PAF (176). The same authors, in a separate *in vivo* study, found that the tenofovir/emtricitabine/efavirenz regimen significantly reduced both the *de novo* and the remodeling of PAF biosynthetic routes in leukocytes, but not in platelets (174). It remains unclear, however, whether the effect of these regimens on PAF are due to the individual ARVs or one or more combinations within a regimen.

It is plausible that the cART regimens which were found to increase PAF levels and metabolism *in vivo* could contribute to inflammation-linked chronic disorders in HIV-infected patients on cART. On the other hand, it is also possible that reduced PAF degradation in blood cells could, in fact, augment inflammation-linked chronic disorders (173, 174). In the context of unclear, inconclusive, and sometimes contradictory, data on PAF biosynthesis, it is important to continue studying the effects of existing, as well as new ARV drugs on the biosynthesis of PAF and its possible effects on HIV pathogenesis, preferably in prospective, randomized studies.

Although platelets possess the necessary machinery to intercept and eradicate single-stranded RNA viruses such as HIV, recent evidence has also implicated these cells in HIV disease pathogenesis and persistence during virally-suppressive therapy. Viral persistence in this setting is associated with ongoing, albeit low-grade, chronic activation of platelets, creating a pro-thrombotic systemic milieu. This, in turn, may be intensified by certain ARVs such as abacavir and ritonavir-boosted lopinavir/darunavir. Although somewhat contentious, anti-platelet agents may be of benefit not only in the prevention of HIV-related CVD in patients who are ostensibly virally-suppressed, but also in depleting residual virus.

The effect of HIV on platelet function, the mechanisms involved and the effects of cART on these functions are summarized in Table 2.

**Table 2:** Effects of HIV-infection and combination antiretroviral therapy on platelets.

**Effector function of platelets**	**Effects of HIV on platelet function**	**Consequence**	**Effect of cART on platelet function**
	Platelet numbers	• Thrombocytopenia as a result of reduced production or destruction (123).	• Correct after initiation of cART (177).
Trap and eliminate pathogens	HIV dissemination	• Platelets may promote HIV-1 spread as they bind to virus via CLEC-2 and DC-SIGN (112). • Significant levels of HIV-1 associated with platelets during all stages of infection (178). • Platelets harbor replication competent HIV (115, 116). • Platelet-CD4^+^ T-lymphocyte complex formation drives CD4^+^ T-lymphocyte infection by platelet-bound virus (116).	• Platelets from HIV-infected individuals on ART with poor CD4+ T cell recovery can harbor HIV despite viral suppression (115, 116).
Platelet activation	Increased	• Upregulation of expression of platelet activation markers, i.e., CD62P and CD40L (127, 130, 134, 135). • Increased spontaneous and activated platelet aggregation; elevated levels of platelet glycoprotein (GP) IV (CD36, a marker of platelet aggregation) (133, 179). • Raised levels of platelet-derived inflammatory mediators including RANTES, CD40L, CD62P, LIGHT, NAP-2 (132). • Increased platelet oxidative stress as measured by activation of NOX2 that may be related to platelet activation (136).	• Levels of sGPVI, sCD40L and sCD62P reduced by cART (TDF/FTC plus NNRTI) (134). • Raltegravir treatment reduced CD62P expression (128). • Raised levels of RANTES, CD62P, LIGHT, and sCD40L were not normalized by cART (132). • Abacavir treatment caused increased platelet reactivity as showed by some (142, 144, 145) but not others (146, 147). • Ritonavir increased production of platelet-derived TGF-β (153, 154) and PGE_2_ (158). • Darunavir increased platelet degranulation (155).
Platelet-leukocyte aggregates	Increased	• Increased platelet-monocyte aggregates (117, 128, 179, 180).	• Platelet-monocyte aggregates reduced by raltegravir treatment (128). • Platelet-leukocyte aggregates (and/or platelet-monocyte complexes) remained elevated in patients on ART (117, 128).
Platelet-endothelial cell interaction	Increased		• Abacavir enhanced interaction (118).

*cART, combination antiretroviral therapy; CD40L, CD40 ligand; CLEC-2, C-type lectin-like receptor 2; DC-SIGN, Dendritic Cell-Specific ICAM3-Grabbing Non-integrin; FTC, emtricitabine; LIGHT, homologous to lymphotoxin, inducible expression, competing for GpD of herpes virus, that binds to the HVEM, and is expressed on activated T lymphocytes; NAP-2, neutrophil activating peptide-2; NNRTI, Non-nucleoside reverse transcriptase inhibitor; NOX2, NADPH oxidase 2; PGE_2_, prostaglandin E_2_; RANTES, Regulated on Activation, Normal T Cell Expressed and Secreted; sGPVI, soluble glycoprotein VI; TDF, tenofovir disoproxil fumarate; TGF-β, transforming growth factor beta*.

## Neutrophil-Platelet-Endothelium Interactions

While the focus of the review is on neutrophils and platelets, it should, however, be mentioned that HIV infection is also associated with endothelial dysfunction, with multiple mechanisms identified, including chronic inflammation, hypercoagulability, increased cell adhesion and platelet activation (143, 181, 182). Combination ART has been shown to have variable effects on endothelial dysfunction in HIV-infected patients with some studies reporting an improvement (181, 183–185), while others found that inflammation and endothelial activation persisted after long-term cART (186).

In response to infection or injury, interactions between neutrophils, platelets, and the endothelium take place through various recruitment signals, adhesion molecules and regulatory pathways, which lead to neutrophil recruitment via rolling and arrest on endothelial cells and, finally, emigration from the vessel, where they fight pathogens and contribute to re-establishment of tissue integrity (187). Interactions between platelets and the endothelium have been extensively reviewed previously (188). Briefly, interactions are mediated by various pathways, the most notable being platelet GPIbα (in the GPIb-IX-V receptor complex) with von Willebrand factor (VWF) released from the endothelium under low shear stress conditions; bi-directional binding of CD62P with PSGL-1 or GP1bα under high shear stress conditions; firm adhesion facilitated by platelet GPIIb/IIIa (integrin αIIbβ3) with endothelial α_v_β_3_ (via VWF, fibrinogen, fibronectin), ICAM-1 (via fibrinogen); and GPIb-IX-V (via VWF) (189, 190). Platelets bound to activated endothelial cells can also lead to recruitment of neutrophils with the interactions between neutrophils and platelets occurring first, followed by neutrophil-endothelial interactions (191). The complex and multi-faceted relationship that exists between these cells (neutrophils, platelets and endothelial cells) have been reviewed in detail elsewhere (191–193). Here we will limit our narrative to a brief overview of the effect of HIV and ART on neutrophil-platelet-endothelium interactions.

### The Effect of HIV on Neutrophil-Platelet-Endothelium Interactions

During inflammatory responses arising from a diverse range of inflammatory, autoimmune and infectious diseases, neutrophils and platelets form neutrophil-platelet (NP) aggregates (NPAs) (193, 194). These NPAs induce increased production of pro-inflammatory molecules and increased chemotaxis of neutrophils to sites of infection. The NP interaction is primarily mediated by the binding of CD62P on platelets to its counter-receptor, PSGL-1, on the neutrophil surface, and platelet GPIbα binding to neutrophil macrophage-1 antigen (MAC-1), a heterodimer consisting of CD11b and CD18 chains (194, 195).

Increased platelet/platelet microparticle activation and expression of surface sCD62P, as well as tissue factor, albeit debatable and seemingly difficult to demonstrate (196, 197), have been observed in HIV-infected individuals. This is possibly due, at least in part, to increased microbial translocation from the gut as these biomarkers have been shown to correlate with the lipopolysaccharide (LPS) receptor, sCD14 (127). The authors of this study concede, however, that this proposed linkage is limited by the fact that they did not measure LPS directly (127). In addition, it has been found that LPS and other bacterial products bind to TLRs resulting in activation of platelets (198). This sustained platelet activation may contribute to the persistent inflammation and cardiovascular morbidities observed in HIV-infected individuals, despite cART (130).

Not only do platelets promote adhesion and transmigration of neutrophils across the endothelium (199), but their interactions with neutrophils also enhance neutrophil defense mechanisms against pathogens. However, unregulated neutrophil reactivity induced by platelets may be associated with the development of various inflammatory disorders that can affect the heart, pancreas, liver, kidney, lung, brain, intestinal tract, and reproductive system (200). These non-AIDS defining diseases are also observed in HIV-infected individuals receiving cART (201).

Another important mechanism driven primarily by NP interactions to restrict infection and eliminate pathogens is NET activation and release (NETosis). NETosis is characterized by the formation of NETs, in which cellular deoxyribonucleic acid (DNA), histones, cytoplasmic granules, and other proteins, such as myeloperoxidase and elastase, are released into the extracellular space where they immobilize and facilitate killing of invading microorganisms (202). Indeed, NETs released by neutrophils in the genital tract have been reported to inactivate HIV-1, thereby preventing infection of “HIV-susceptible cells” (88). Sivanandham et al. also found that in an attempt to phagocytose microbes released during translocation, neutrophils are driven to the formation of NETs (87). Not only do NETs capture and inactivate SIV/HIV but they also trap CD4+ T-lymphocytes, CD8+ T-lymphocytes, B-lymphocytes, and monocytes, potentially contributing to an indiscriminate generalized immune cell loss (87). In addition, platelets are captured in NETs resulting in platelet aggregates, which could promote thrombosis due to the expression of tissue factor (203). Immune failure in these individuals, despite treatment with cART, as well as other non-AIDS defining comorbidities associated with SIV/HIV infection, may also be exacerbated by the excessive intravascular NETosis observed in these individuals (87). On the other hand, NETosis has been reported to be inhibited by the binding of HIV-1 to DCs with the subsequent production of IL-10 by these cells (89). The IL-10 produced by the DCs inhibits ROS-mediated NET formation, resulting in impaired HIV elimination. However, IL-10 production by DCs only occurs 3 h post-stimulation with HIV, while NETosis takes place rapidly (88).

Importantly, unregulated and prolonged neutrophil reactivity induced by activated platelets may be associated with the development of various inflammatory disorders, including cardiovascular conditions and rheumatoid arthritis (204–206). In the setting of HIV-infection, persistent activation of immune cells, despite cART (207), has been reported to contribute to the increase in inflammatory disorders observed in these individuals (208, 209).

HIV infection further stimulates platelet-endothelium interactions with elevated levels of sVCAM-1, sICAM-1, and VWF observed in HIV-infected persons (210, 211). These markers have been associated with chronic endothelial activation, atherosclerosis and thrombosis, culminating in increased risk of CVD. A discussion of immune-thrombosis is beyond the scope of this article and the role of platelets in thrombosis has been reviewed extensively by Koupenova et al. (212, 213)

### Effects of Antiretroviral Agents on Neutrophil-Platelet-Endothelium Interactions

#### Nucleos(t)ide and Non-nucleoside Reverse Transcriptase Inhibitors

Although the study by Alvarez et al. could not confirm the stimulatory effects of abacavir on platelets, these authors did, however, report that abacavir promoted adherence of platelets to endothelial cells, a process regarded as a key step in thrombus formation (147). In these experiments, platelets were drawn across a human umbilical vein endothelial cell (HUVEC) monolayer in the absence and presence of test ARVs. Only abacavir, but none of the other NRTIs investigated, was shown to induce a significant, dose-dependent increase in the number of platelets adhering to endothelial cells (147). “This resulted from activation of the endothelium via purinergic ATP-P2X_7_ receptors, which subsequently triggered the interplay of specific adhesion molecules located on both cell populations: namely, sCD62P and ICAM-1 on endothelium interacting with GPIIb/IIIa and GPIbα on platelets” (147, 214). Furthermore, it was shown that abacavir, but not tenofovir, dose-dependently increased thrombus formation in an animal model, by ATP-P2X_7_ receptor activation (215). These findings suggest that abacavir induces a pro-inflammatory vascular environment, which may provide a mechanistic explanation for some of the cardiovascular actions of this NRTI (147).

An increase in vascular permeability coupled with leukocyte infiltration is a hallmark of the inflammation that underlies vascular diseases, as well as HIV infection *per se* (216). Somewhat similarly to the study reported by Alvarez et al., also using an *in vitro* model in which leukocytes, as opposed to platelets, flow over a monolayer of HUVECs, abacavir was shown to induce leukocyte accumulation by increasing neutrophil and PBMC (peripheral blood mononuclear cells) rolling and adhesion (217). In these experiments, abacavir enhanced the interaction between leukocytes and endothelial cells by activating the neutrophil adhesion molecule, Mac-1(CD11b/CD18), which, in turn, interacts with ICAM-1 on endothelial cells (217). This effect was reproduced by didanosine (another NRTI that has been implicated in a raised risk of MI), as well as the NNRTIs, efavirenz and nevirapine, but not by other NRTIs or the PI, lopinavir (216–218).

#### Protease Inhibitors

While no prospective, randomized trials have been performed to determine a causative relationship between ARV agents and CVD (157), observational studies have reported an association between an increased risk of acute MI (AMI) and cumulative exposure to ritonavir-boosted lopinavir or darunavir. The reasons for this are still poorly understood and may be confounded by PI-induced off-target metabolic effects. It has also been suggested that increased CVD risk could be aggravated by drug-induced endothelium injury. For instance, *in vitro* work demonstrated that ritonavir directly causes endothelial mitochondrial DNA damage and cell death through necrosis pathways (219).

In contrast, some studies have suggested that atazanavir may effect a reduction in AMI risk (220). The reasons why atazanavir appears to be the exception among the PIs remain speculative, but have been proposed to be related to improved endothelial function, possibly secondary to elevated bilirubin levels. Bilirubin is known to be a powerful endogenous antioxidant, a property, which has been proposed to be of clinical importance (221, 222). In fact, a recent study among HIV-infected individuals in the Veterans Aging Cohort Study demonstrated an inverse association between bilirubin and total CVD, AMI, heart failure, and ischemic stroke events after adjusting for known risk factors (223). These findings are supported by a small double-blind, randomized crossover study of 15 patients with known type 2 diabetes mellitus who were treated with atazanavir for 3 days, followed by a 3-days placebo treatment. While on atazanavir, patients had improved antioxidant capacity, as measured by the ferric reducing ability of plasma assays, and lower levels of vascular inflammation, as indicated by decreased plasma levels of von Willebrand factor, while those of soluble vascular cell adhesion molecule-1 (sVCAM-1) and sICAM-1 were not affected, and the plasma levels of fasting glucose and LDL cholesterol were also unchanged (224).

#### Integrase Strand Transfer Inhibitors

Despite hopes for improved CVD outcomes on INSTIs, observational studies suggest that switching from PI-based ART to a raltegravir-based regimen may not reduce platelet activation (160, 161) or arterial inflammation (225). Martinez et al. demonstrated that markers of endothelial activation, namely sICAM-1, sVCAM-1, E-selectin, and sCD62P, did not improve on the INSTI.

Recent reports have highlighted the pro-thrombotic role of NETs, however, it appears that no studies have investigated the impact of cART, either alone or in combination, on NET production during HIV infection (87). A single study in 12 SIV-infected non-human primates reported that co-formulated, unspecified, virally suppressive ART decreased, but did not normalize, NETosis after 10 months of treatment (87).

The aforementioned studies are seemingly consistent with the involvement of platelet/neutrophil/vascular endothelial interactions in establishing a labile intravascular milieu, possibly driven by microbial translocation. This situation is evident not only in the setting of untreated HIV infection, but also during virally-suppressive therapy, possibly exacerbated by agents such as abacavir and ritonavir-boosted lopinavir/darunavir. Given the potential role of persistent intracellular viral reservoirs in fueling this pro-inflammatory environment, future research should focus on identifying combinations of ARVs that are most effective in targeting these viral populations.

## Conclusion

This review focused on the role of neutrophils and platelets in HIV transmission and disease and explored the effect of HIV and the most common ARV agents on the numbers and functions of neutrophils and platelets, as well as on neutrophil-platelet interactions. While many individual ARVs were discussed in terms of detrimental effects on neutrophil or platelet function, it is difficult to draw concrete conclusions from *in vitro* studies on individual ARVs due to the *in vitro* experimental set-up differing substantially from the therapeutic setting in humans on cART. In addition, a single ARV agent may have both positive and negative effects on the pathophysiological pathways under study, obscuring the clinical consequences of combination drug use. Nevertheless, individual ARV agents, such as abacavir, lopinavir, and darunavir, have been associated with an increased risk of CVD in clinical studies and *in vitro* work has provided insight into possible mechanistic pathways underlying this risk. The long-term impact of INSTIs on immune cells is still poorly understood and should be closely monitored since this has become the regimen of choice in all international HIV treatment guidelines. In light of the importance of CVD and HAND in the long-term health and quality of life of people living with HIV, further prospective studies with various drug combinations assessing clinically relevant endpoints are needed.

## Author Contributions

All authors listed have made a substantial, direct and intellectual contribution to the work, and approved it for publication.

## Conflict of Interest

The authors declare that the research was conducted in the absence of any commercial or financial relationships that could be construed as a potential conflict of interest.
